# A longer Achilles tendon moment arm length is not associated with superior hopping performance

**DOI:** 10.3389/fbioe.2023.1270169

**Published:** 2023-10-26

**Authors:** Bálint Kovács, Sun Dong, Yang Song, Ye Jingyi, Sándor Béres, József Tihanyi, Jingfeng Zhang, Leonidas Petridis, Yaodong Gu

**Affiliations:** ^1^ Faculty of Sport Science, Ningbo University, Ningbo, China; ^2^ Department of Kinesiology, Hungarian University of Sports Sciences, Budapest, Hungary; ^3^ Department of Athletics, Hungarian University of Sports Sciences, Budapest, Hungary; ^4^ Department of Radiology, HwaMei Hospital, University of Chinese Academy of Sciences, Ningbo, China; ^5^ Research Centre for Sport Physiology, Hungarian University of Sport Sciences, Budapest, Hungary

**Keywords:** hopping, muscle structure, moment arm, lower leg, triceps surae muscle

## Abstract

Variability in musculoskeletal and lower leg structure has the potential to influence hopping height. Achilles tendon moment arm length and plantarflexor muscle strength can influence ankle joint torque development and, consequently, hopping performance. While most studies have examined the connection of the Achilles tendon moment arm with hopping performance including the resting length, in this study we attempted to explore how the changes in Achilles tendon moment arm are related to hopping performance. Therefore, the purpose of this study was to test for correlations between foot and lower leg muscle structure parameters (i.e., muscle mass, volume, cross-sectional area and Achilles tendon moment arm length) and hopping height performance in relation to changes in Achilles tendon moment arm length. Eighteen participants (10 males 8 female) performed repetitive bilateral hopping on a force platform while sagittal plane kinematics of the lower leg were recorded. Additionally, maximal isometric plantarflexion was measured. To obtain structural parameters of the lower leg, the right lower leg of each participant was scanned with magnetic resonance imaging. The cross-sectional areas of the Achilles tendon, soleus, lateral and medial gastrocnemius were measured, while muscle volumes, muscle mass, and Achilles tendon moment arm length were calculated. Contrary to our initial assumption, longer Achilles tendon moment arm did not result in superior hopping performance. Interestingly, neither maximal isometric plantarflexion force nor muscle size correlated with repetitive bilateral hopping performance. We can assume that the mechanical characteristics of the tendon and the effective utilization of the stored strain energy in the tendon may play a more important role in repetitive hopping than the structural parameters of the lower leg.

## 1 Introduction

Ankle joint torque is the product of the applied muscle force and the length of the Achilles tendon moment arm (ATMA). Accordingly, this structural attribute is of great importance in joint torque development (Piazza et al., 2011; Baxter et al., 2012; Baxter and Piazza, 2014). The resting ATMA length is genetically determined (Raichlen et al., 2011), but the dynamic ATMA length changes with ankle joint angular displacement (Fath et al., 2013; Deforth et al., 2019), which in turn may modify the mechanical capability of the plantarflexor muscles (Dick et al., 2017; Monte et al., 2021). Several studies have investigated how length changes in ATMA during joint rotation influence muscle output (Nagano and Komura, 2003; Harkness-Armstrong et al., 2020; Monte et al., 2021). In a model simulation, Nagano and Komura (2003) reported that a longer ATMA generated higher joint torque during eccentric contraction (<120 deg∙s^-1^), while during concentric contraction, higher joint torque was produced with a shorter ATMA compared to a longer ATMA. In contrast to these results, Baxter and Piazza (Baxter and Piazza, 2014) reported greater maximal ankle torque with a longer ATMA during both isometric and isokinetic contractions. In the latter study, the volume of the plantarflexor muscles was also assessed, showing only a moderate correlation with both isometric and isokinetic peak ankle joint torque, indicating that the muscle volume alone may not be a major influencing factor in joint torque development.

A few studies have attempted to explore the influence of ATMA on the bilateral hopping task. During hopping, the ankle joint absorbs and generates the largest amount of power among the lower limb joints, and the movement occurs mainly in the vertical direction (Farris and Sawicki, 2012). Werkhoven and Piazza (2017b) found that a higher jumping height in a single-joint jumping task can be achieved with a longer heel length, which could indirectly indicate a positive connection between jumping height and a longer ATMA. Kenyan runners possess a longer ATMA (Kunimasa et al., 2014) and hop higher (Sano et al., 2013) compared to Japanese long-distance runners. However, the connection between these variables was not investigated, thus we cannot conclude that superior hopping performance is related to a longer ATMA.

In addition to ATMA, the external moment arm and the ratio of ATMA to the external moment arm have been shown to influence the functional role of the ankle, which primarily operates as a spring during hopping (Monte et al., 2021). Therefore, the ratio of ATMA to the lever arm length (external moment arm) can reflect the effective mechanical advantage (Harkness-Armstrong et al., 2020; Monte et al., 2021), which can explain the magnitude of force production required to achieve hopping height.

Ankle joint torque also depends on the activation of the plantarflexor muscles and their force generation capacity, which is closely related to the muscles’ structural characteristics. Generally, larger muscle size (i.e., volume, mass, anatomical cross-sectional area, etc.) is associated with greater force output in the plantarflexor muscles (Bamman et al., 2000; Baxter and Piazza, 2014), which presumably could lead to a greater hopping height. In addition to muscle characteristics, tendon properties could also influence hopping performance (Lamontagne and Kennedy, 2013). Collectively, triceps surae muscle mass, plantarflexor strength capacity, and the ATMA all contribute to hopping performance. However, considering that the dynamic length changes of the ATMA seem to influence ankle joint function and force generation capacity, it is important to explore how muscle dimensions are linked to joint torque in relation to changes in ATMA length. Exploring the connection between interindividual variation in human lower leg muscles and foot structure with hopping performance will improve our understanding of the mechanisms behind the association of structural and functional indices and may provide better insight into the determinants of hopping performance. Therefore, the purpose of this study was to test for correlations between lower leg and foot structural measures such as muscle mass, cross-sectional area and ATMA length and the mechanical characteristics of hopping such as hopping height, ground reaction force and ankle joint kinematics measured during an ankle thrusted bilateral hopping task. Given that ankle joint moment is the product of muscle force and muscle moment arm, we hypothesized that individuals with a longer ATMA would be able to produce higher ankle joint moments that accelerate the center of mass of the whole-body therefore, achieve greater hopping height.

## 2 Materials and methods

### 2.1 Participants

Sample size calculations (G*Power 3.1.7) (Faul et al., 2009) revealed that a minimum sample size of 17 participants would be appropriate to detect significant correlation between ATMA length and hopping height, assuming a moderate effect size, type I error of 0.05, and power of 0.80. Eighteen healthy, physically active young adults (10 males, 8 females) volunteered to participate in the study. The participants had an average age of 26.4 ± 4.1 years, body height of 1.74 ± 0.08 m, and body mass of 63.2 ± 6.5 kg. They regularly exercised at least three times per week and had no history of neurological or musculoskeletal injuries in the lower extremities over the past 2 years. Prior to the experiments, participants were provided with information about the experimental protocol, and they provided written informed consent to participate in the study. The study was conducted in accordance with the Declaration of Helsinki and was approved by the ethical committee of the Hungarian University of Sports Science (TE-KEB/13/2022).

### 2.2 Experimental protocol

The participants visited the lab on two separate occasions. During the first visit, the hopping test was conducted. Upon arrival, participants performed a standardized warm-up protocol, which included 5 min of cycling on an ergometer, dynamic stretching, and submaximal jumps to ensure proper technique. Following the warm-up, four reflective markers with a diameter of 1 cm were attached to the right leg of each participant at the following locations: the greater trochanter of the hip, lateral condyle of the tibia, lateral malleolus of the ankle, and the fifth metatarsal head of the foot. These markers were used to estimate ankle and knee joint angular displacement during the hops. The participants then performed several minutes of bilateral hops while wearing the equipment. The bilateral hops were conducted barefoot on a force platform, with arms on the hips. Participants were instructed to gradually increase the height of their hops from low to maximum, ensuring that at least three stable maximal hops were recorded (Sano et al., 2013). The three best hops, determined by the reactive strength index (the ratio of flight to contact time), were included in the analysis. The participants were directed to hop with maximal effort, maintain active plantar flexion, and limit knee and hip flexion throughout the hops. The experimenter monitored the trials, and if any hops were executed incorrectly (with high magnitude of knee and/or hip motion), the trial was repeated. Ankle and knee joint angular displacement and ground reaction force (GRF) were recorded simultaneously during the hops. All devices were synchronized using a custom built three channel synchronization unit.

Prior to the hopping test the maximum voluntary isometric plantarflexion contractions (MVIC) were performed to determine the force generation capacity of the plantarflexor muscles. The participants stood upright with their feet parallel and ankles in a neutral position on a force platform surrounded by a metal frame (Figure 2). A metal rod was placed on the participants’ shoulders and securely fixed to the frame to prevent any movement of the rod. The participants were then instructed to push against the rod with the ball of their feet as forcefully as possible while maintaining an extended torso, hips, and knees. The fixation of the rod minimized ankle angular displacement during the MVICs. Due to the compliance of this system, the ankles were approximately in 100° of plantarflexion (with neutral position being 90°) at the peak ground reaction force. The MVICs were performed bilaterally, similar to the way the hops were conducted. Three MVICs were carried out with at least 2 minute rest period in between, and the best trial was included in the analysis.

On the second occasion participants come to a hospital to measure the anatomical characteristics of the triceps surae muscle-tendon complex, where Magnetic Resonance Imaging (MRI) scans were taken of the right leg.

### 2.3 Measurement of the kinematic and kinetic parameters

Hops were performed on a force plate (Kistler Force Platform System 92-81B, Switzerland) with a sampling frequency of 1,000 Hz. The raw ground reaction force data was filtered using a fourth-order low pass Butterworth digital filter with a cut off frequency of 50 Hz (Barker et al., 2017). Contact and flight times, as well as mechanical variables, were determined from the force time data. The initial touch down and take-off instants were determined by the GRF, using a threshold of 30 N. The data from the three processed hops were time normalized and averaged.

Hops were recorded at 120 Hz using a digital camera (Sony IMX240), which was positioned 4 m from the force platform on a tripod at a height of 1 m. The recorded video footage was imported and analyzed using Skillspector software (v. 1.2.4, Denmark). The subphases of hopping, namely, the braking and take-off phases, were determined based on the ankle and knee angular displacement time curve. The braking phase was defined as the period between the initial foot contact and the point of most dorsiflexed ankle angle, while the push off phase was defined as the period between the lowest ankle angle and toe-off. A neutral knee joint angle of 180⁰ was considered when the longitudinal axes of the thigh and shank were aligned, and a neutral ankle joint angle of 90⁰ was considered when the shank was perpendicular to the foot base (sole).

### 2.4 MRI measurements

The anatomical characteristics of the triceps surae muscle-tendon complex were assessed on a separate day using MRI. T1-weighted MRI scans were taken on the right leg with a 3T Philips scanner (Ingenia 3.0T MRI system, Amsterdam, Netherlands). Participants were positioned in a supine position with neutral knee and ankle joint angles. To avoid weight induced deformation of the muscle during the scan, a 10 cm tall foam pad was placed below the calcaneus to slightly elevate the leg. Scans started distally from below the calcaneus to approximately to the middle of the SOL muscle. Then from this point another scan was taken to the epicondyles of the knee where MG and LG muscles originate. The scans were performed using a T1-weighted turbo spin echo sequence with the following parameters: slice thickness = 5 mm, slice gap = 0 mm, slice scan order: interleaved, TR = 650 m, TE = 20. Due to the limited field of view of the probe (40 cm), the scans had to be acquired in two parts to ensure that the image sequence captured the origin and insertion of the plantar flexor muscle-tendon complex. The scanning axes were carefully aligned with the muscle-tendon unit by a qualified technician.

The length of the ATMA was estimated from sagittal plane MRI images. During this scan, participants lay on their sides, and the right ankle was set to a neutral angle joint. The ankle joint angle was measured with a goniometer to ensure consistent joint position for all participants. To prevent any movement during the scans, the ankle was secured with medical sandbags. The scans were repeated with the ankle set at a 15° plantar flexion angle joint.

**FIGURE 1:**
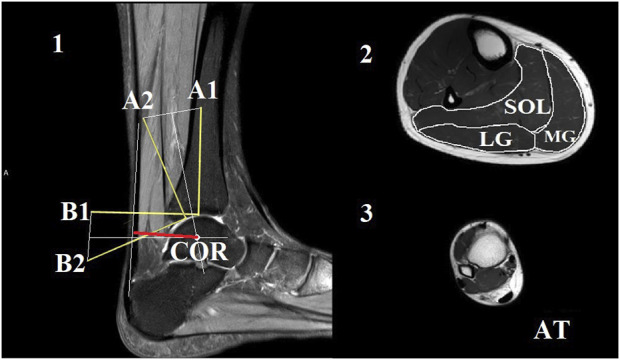
The Reuleaux graphical method was used to estimate the location of the center of rotation (COR) at the neutral joint position [1]. In this method, the talus was considered a fixed location, and the plantarflexion of the ankle was represented by the movement of the tibia with markers A1 and B1 to the next joint position marked with A2 and B2, respectively. Perpendicular lines were drawn to the bisectors of the connecting lines of A1-A2 and B1-B2. The intersection of these perpendicular lines marked the COR. The line of action for the Achilles tendon was marked in the image (indicated by the orange line). The length of the perpendicular line between the COR and the action line of the Achilles tendon (indicated by the red line) was considered as the length of the Achilles tendon moment arm. Representative magnetic resonance images from the middle of the lower leg were used for the calculation of cross-sectional areas [2]. The soleus (SOL), medial gastrocnemius (MG), and lateral gastrocnemius (LG) compartments were outlined manually. Additionally, a sample image of the maximal distal Achilles tendon (AT) was included [3].

The axial plane images were analyzed using ImageJ 1.44b software (National Institutes of Health, United States). The cross-sectional area (CSA) of each muscle and the Achilles tendon was manually outlined and measured (Figure 1). To ensure the reliability of the CSA measurements, the images were analyzed by two individuals. The average CSA measured by the two analysts was used to calculate muscle volume and mass. Muscle and tendon lengths were calculated by summing the number of analyzed slices and multiplying by 0.5. The volume of each muscle was calculated by summing the volume of each slice, which is the product of the cross-sectional area and slice thickness (5 mm) (Kovács et al., 2020). Muscle mass was then calculated as the muscle volume multiplied by muscle density (1.056 g/cm³) (Winter 2009).

### 2.5 Estimation of Achilles tendon moment arm length

We utilized the Reuleaux graphical analysis (König, 2015) to estimate the length of the ATMA by characterizing the ankle joint with the tibiotalar joint (Figure 1). In the first step, a line (L1) was drawn along the longitudinal axis of the tibia, starting from the most distal articular surface of the bone in the image taken at the neutral ankle joint position. Then, a point (A1) was marked 7 cm from the starting point of this line. Subsequently, a perpendicular line (L2) was drawn from the same starting point. Similarly, a point (B1) was placed 7 cm from the starting point on L2. The same process was repeated on the image taken from the ankle in a 15° plantar flexed position to determine points A2 and B2. The two images were then overlapped in such a way that the two talus bones aligned as closely as possible with each other. Points A1 and A2, as well as points B1 and B2, were connected by straight lines (La and Lb). Next, two perpendicular lines were drawn from the bisectors of La and Lb, and the intersection of these lines was marked as the center of rotation. The Achilles tendon action line was identified at the 90° ankle joint position as a straight line. The ATMA length was determined as the shortest perpendicular distance from the center of rotation to the tendon action line. Additionally, ATMA was normalized to body height due to the high inter individual variation, which ranged from 1.54 m to 1.92 m. To account for the joint dependent length characteristics of the ATMA, we estimated the dynamic ATMA length using a similar approach to that employed by Werkhoven and Piazza (2017a). We utilized experimentally measured ATMA data at different joint angles from a previous study conducted by Maganaris et al. (2000). First, we fitted a second order polynomial line to the original dataset. Next, we used this model to estimate the changes in dynamic moment arm length based on the ankle angle joint displacement data obtained from each participant in our study. To ensure compatibility with our data, the dataset was interpolated over the ankle angle joint displacement data. Finally, the estimated dynamic moment arm lengths were multiplied by a scaling factor. The scaling factor was determined as the measured ATMA length at zero degrees (ankle in a neutral position) obtained from the MRI images.

Foot length was calculated using a sagittal plane photographic image taken prior to the hopping test. Participants were seated with the right foot placed on a reference block, ensuring that the ankle was in a neutral position. The foot was then photographed from both the lateral and medial sides in the sagittal plane. The distance from the most prominent tip of the first hallux to the posterior aspect of the Achilles tendon was measured on both sides. The mean of these two distances was taken as the foot length. The gear ratio was calculated as the ratio of the foot length to the moment arm length (Fletcher and MacIntosh, 2017).

### 2.6 Statistical analysis

Data are presented as mean and standard deviation (±SD). The distribution of the data was checked with Shapiro–Wilk test. To determine the relative between rater reliability of each muscle and tendon, an intraclass correlation coefficient (ICC) was calculated using a two-way mixed effects model (average measures), along with the upper and lower 95% confidence interval (CI±). The ICC estimate was considered good between 0.75 and 0.9 and excellent above 0.9 (Koo and Li, 2016). A Bland–Altman plot was used to determine the bias between the conductors and the limits of agreement (see Supplementary Material). Relationship between variables was estimated with Pearson’s correlation coefficients (r), which was categorized as small (0.0–0.1), moderate (0.1–0.3), medium (0.3–0.5), large (0.5–0.7), and very large (0.9–1.0) (Hopkins et al., 2009). Additionally, the 95% confidence intervals for each coefficient were calculated. The level of significance was set at *p* ≤ 0.05.

## 3 Results

**FIGURE 2:**
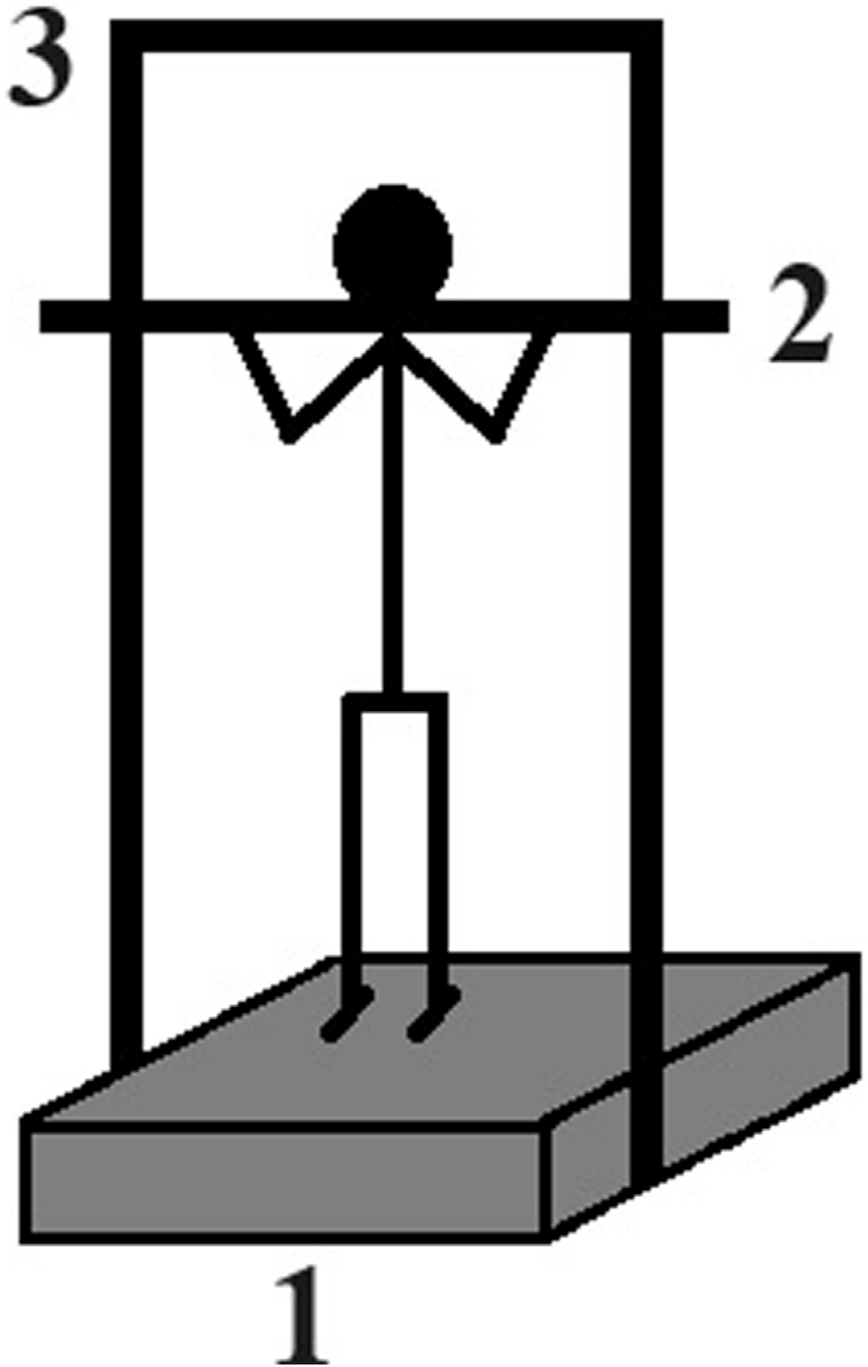
Schematic figure of the maximal isometric strength test of the plantarflexor muscles. Participant stood erect on a force plate (1) and a metal frame (3) was fixed around the force plate. A metal rod (2) was placed on the shoulder of the participants which was fixed to the metal frame. Participants were asked to push the rod upward as forcefully as they can with straight legs and hip without any trunk movement while vertical ground reaction force was recorded.

**FIGURE 3:**
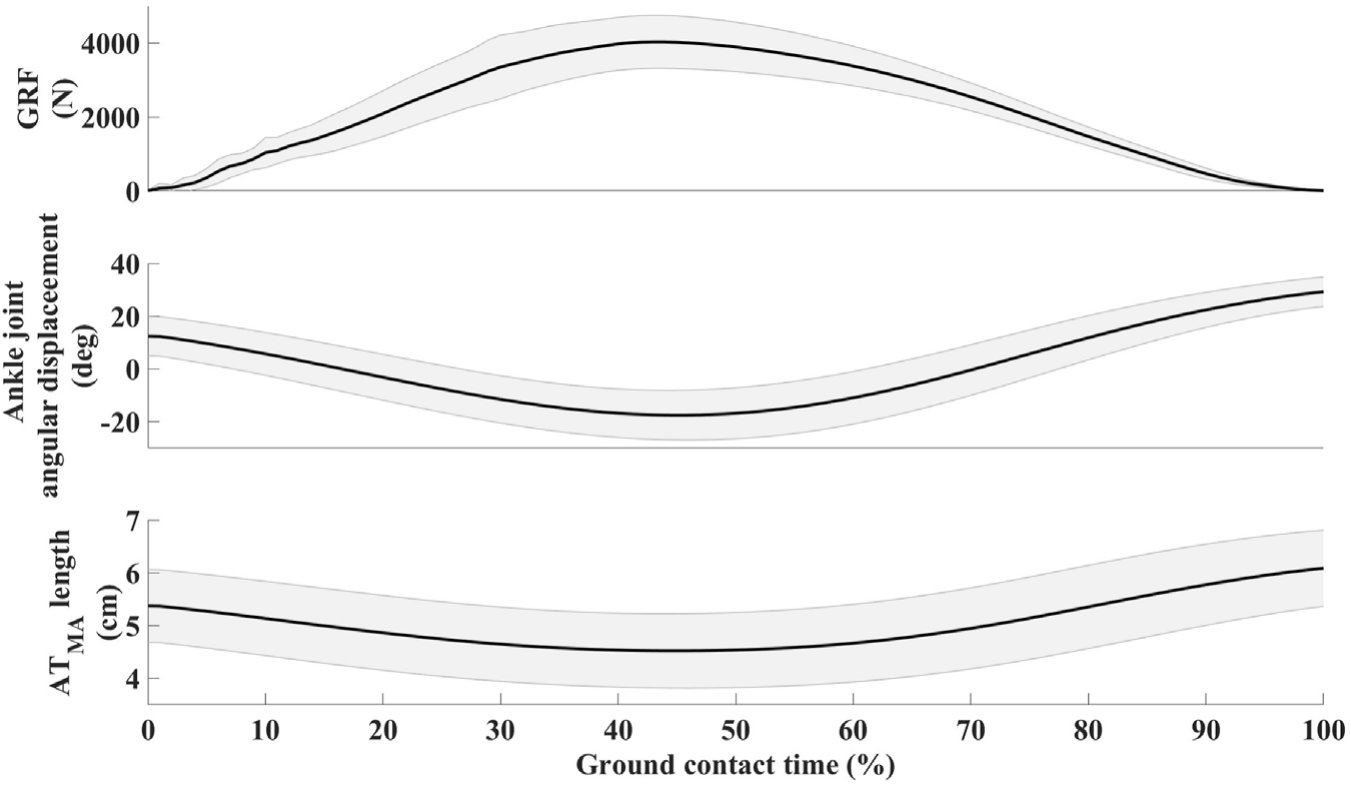
Mean ± SD of vertical ground reaction force (GRF), ankle joint angular displacement, and Achilles tendon moment arm (ATMA) length changes in relation overground contact.

**FIGURE 4:**
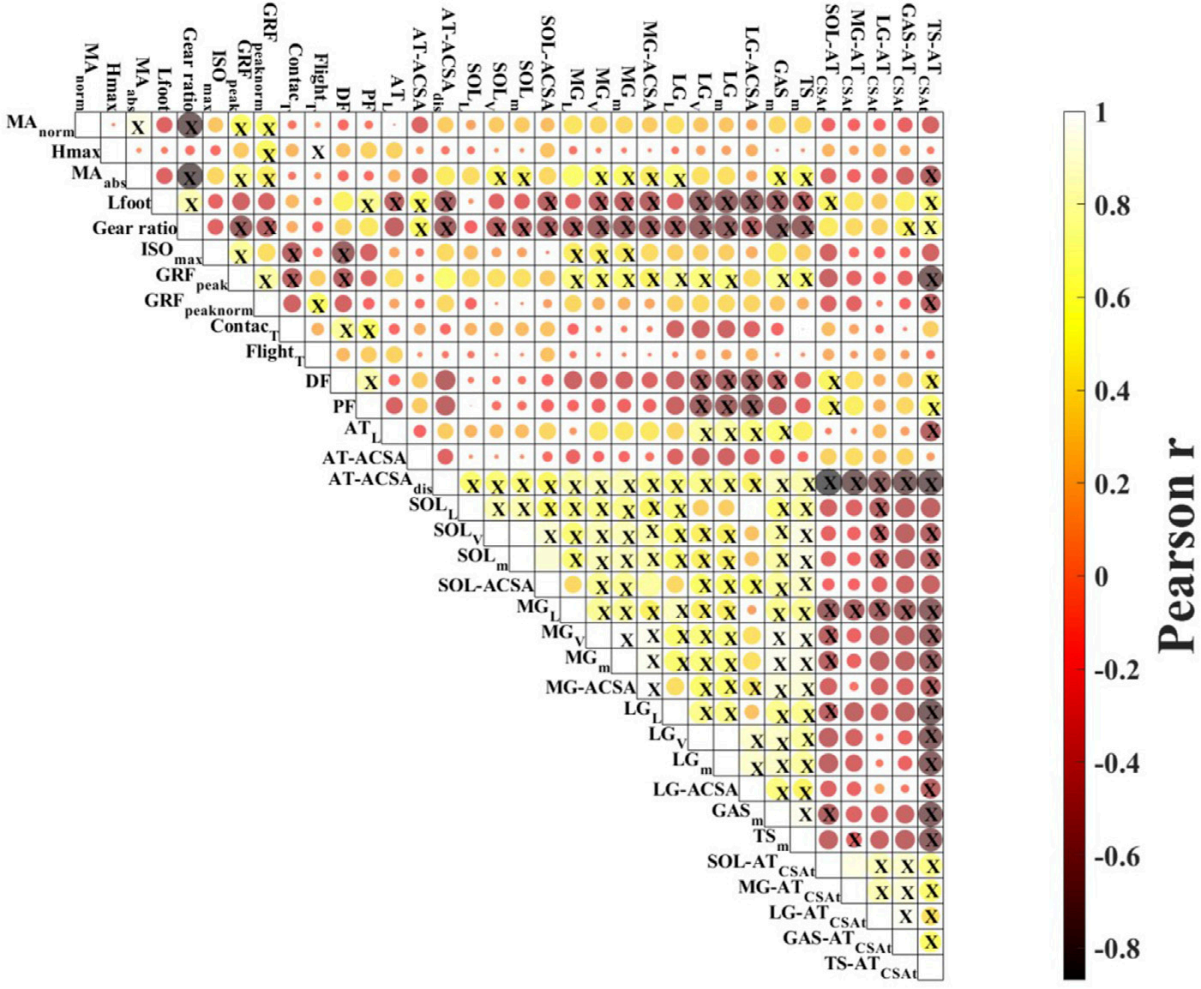
Correlation matrix plot. Results of the Pearson correlation analysis. Yellow circles show positive and red circles negative correlations; colour saturation is proportional to the magnitude of the correlation. X in the circle indicates significant correlations (*p* < 0.05). Abbreviation: MAnorm—normalized Achilles tendon moment arm; Hmax - peak hopping height; MAabs—absolute moment arm length; Lfoot—foot length; ISO—maximal isometric plantarflexion; GRFpeak—peak ground reaction force during hopping; GRFnorm—body weight normalized peak ground reaction force; Contactt—contact time; Flightt—flight time; DF—dorsiflexion; PF—plantarflexion; ATL—Achilles tendon length; AT-ACSA—Achilles tendon peak proximal cross-sectional area; AT-ACSAdis—Achilles tendon peak distal cross-sectional area; SOLL—soleus length; SOLV—soleus volume; SOLm—soleus mass; SOL-ACSA—soleus maximal cross-sectional area; MGL—medial gastrocnemius length; MGV—medial gastrocnemius volume; MGm—medial gastrocnemius mass; MG-ACSA—medial gastrocnemius maximal cross-sectional area; LGL—lateral gastrocnemius length; LGV—lateral gastrocnemius volume; LGm—lateral gastrocnemius mass; LG-ACSA—lateral gastrocnemius maximal cross-sectional area; GASm–gastrocnemii muscle mass; TSm—triceps surae muscle mass; SOL-ATCSAt–soleus to Achilles tendon cross-sectional ratio; MG-ATCSAt–medial gastrocnemius to Achilles tendon cross-sectional ratio; LG-ATCSAt–lateral gastrocnemius to Achilles tendon cross-sectional ratio; TS-ATCSAt—triceps surae to Achilles tendon cross-sectional ratio.

The average hopping height was 21.8 ± 4.1 cm. The mean resting length of the ATMA, as measured from the MRI images, was 4.91 ± 0.63 cm (Table 1.). The dynamic length changes of the ATMA during hopping are presented in Figure 2. The minimum length (4.50 ± 0.72 cm) occurred at the midpoint of the jump, around the peak ankle dorsiflexion, while the maximum length (6.08 ± 0.74 cm) (23.6% longer compared to the resting length) was reached at the moment of takeoff. Participants landed with a plantarflexed ankle position (12.13 ± 7.75°) and a slightly flexed knee (171.30 ± 8.04°), resulting in a 9.04% ± 0.06% (5.37 ± 0.71 cm) longer ATMA than the resting length (Figure 3). The reliability test demonstrated excellent agreement between the analysts for each muscle and tendon (ICC >0.97, see in Supplementary Material). The mean and SD values of the plantar flexor muscle tendon unit properties in Table 2. SOL volume was 51.16% of the total triceps sure while MG was 30.44%, and LG was 18.41% respectively. The detailed correlation analysis results are presented in Figure 4 and in the Supplementary Material. None of the morphological parameters (such as volume, cross-sectional area (CSA), and total CSA (tCSA) of the plantarflexor muscle-tendon unit showed any correlation with hopping height. Isometric force was only associated with the length, volume, and mass of the medial gastrocnemius (MG) muscle (Figure 4). Normalized and absolute ATMA length was only correlated with peak ground reaction force (GRF) during hopping. However, there was a correlation between absolute ATMA length and the total mass of the triceps surae muscle group, and it also correlated with peak GRF. Peak GRF was also associated with the MG, lateral gastrocnemius (LG), soleus (SOL), and the total mass of the triceps surae muscles, as well as with hopping height and isometric force. However, when GRF was normalized to body mass, these connections disappeared. Foot length and gear ratio showed negative correlations with most of the muscle characteristic variables.

**TABLE 1:** Descriptive results of the main biomechanical variables of hopping, presented as mean ± SD.

Measured variables	mean ± SD
Achilles tendon moment arm (cm)	4.91 ± 0.63
Normalized Achilles tendon moment arm	0.0028 ± 0.003
Δ- moment arm shortening (cm)	0.87 ± 0.35
Δ+ moment arm lengthening (cm)	1.58 ± 0.37
Gear ratio	4.12 ± 0.93
Maximal isometric plantarflexion (N)	1,398.1 ± 375.8
Peak ground reaction force (N)	4,125.3 ± 660.5
Contact time (s)	0.19 ± 0.03
Flight time (s)	0.42 ± 0.04
Ankle dorsiflexion (deg)	30.4 ± 12.5
Ankle plantarflexion (deg)	47.8 ± 11.9
Knee flexion (deg)	19.6 ± 9.2
Knee extension (deg)	38.8 ± 5.9

**TABLE 2:** The calculated (mean ± SD) structural parameters of the triceps surae muscle–tendon unit.

Muscle, tendon	Dimension/unit	Mean SD
Achilles tendon (AT)	Length (cm)	20.39 ± 3.28
	ACSAdistalmax (cm2)	1.01 ± 0.38
Soleus (SOL)	Length (cm)	32.06 ± 2.72
	Volume (cm3)	412.11 ± 93.00
	Mass (g)	435.19 ± 98.21
	ACSAmax (cm2)	24.64 ± 4.55
Medial gastrocnemius (MG)	Length (cm)	24.75 ± 2.99
	Volume (cm3)	245.20 ± 80.90
	Mass (g)	258.93 ± 85.43
	ACSAmax (cm2)	16.46 ± 4.26
Lateral gastrocnemius (LG)	Length (cm)	22.76 ± 3.25
	Volume (cm3)	148.28 ± 47.37
	Mass (g)	156.59 ± 50.02
	ACSAmax (cm2)	11.38 ± 3.12
Gastrocnemii (GAS)	Mass (g)	406.81 ± 134.13
Triceps Surae (TS)	Mass (g)	842.00 ± 220.64
Muscle to tendon anatomical cross-sectional ratio (tCSA)	SOL- AT	28.04 ± 11.53
	MG-AT	18.26 ± 7.05
	LG-AT	12.95 ± 5.97
	GAS-AT	31.89 ± 12.16
	TS-AT	68.13 ± 45.61

Abbreviation: ACSAmax; maximal anatomical cross-sectional area, CSAdistalmax; distal Achilles tendon maximal cross-sectional area.

## 4 Discussion

The aim of this study was to investigate correlations between lower leg and foot structural measures and hopping performance. Our initial hypothesis, suggesting a correlation between hopping height and ATMA length, was not supported. However, it appears that ATMA is associated with mechanical and morphological parameters that serve as strong indicators of improved hopping performance. Interestingly, none of the structural parameters of the plantarflexor muscles showed a correlation with hopping height.

Our results indicate that the length of the ATMA, whether at rest or during hopping, is not a strong predictor of hopping height (Figure 4). This finding holds true regardless of the applied normalization methods, suggesting that ATMA length may not be a useful indicator of ankle muscle function during hopping. In this study, we used a bilateral hopping technique, similar to previous experiments (Hobara et al., 2007; 2008; Sano et al., 2013), and the hopping height achieved by our participants were consistent with earlier reports (Hobara et al., 2007; 2008; Sano et al., 2013; Kovács et al., 2021a). The mean ATMA length of 4.91 cm observed in our study aligns with findings reported in the existing literature (Fath et al., 2010; Baxter and Piazza, 2014; Deforth et al., 2019; Kovács et al., 2021b), as do the measured and calculated morphological parameters of the plantarflexor muscle tendon unit (Friederich and Brand, 1990; Fukunaga et al., 1992; Baxter and Piazza, 2014; Kovács et al., 2020).

Previous studies have provided conflicting findings regarding the role of ATMA on hopping performance. Watanabe et al. (2008) demonstrated that volleyball players with shorter ATMA were able to achieve higher hops compared to distance runners, although no correlation was observed. In cases where the hop was performed as a static jump (only pushing off from the ground), heel length showed a strong correlation with hop height, suggesting that a longer ATMA may be advantageous for superior hopping performance. In our recent study (2021a), we did not find a direct correlation between ATMA and hopping height. However, it is worth noting that in that study, ATMA length was estimated using a photograph based method, which is considered less accurate (Fath et al., 2010) and may limit the interpretation of the results. On the other hand, we did find a correlation between ATMA length and both absolute and body weight normalized peak ground reaction force (Figure 4). A longer ATMA should, by definition, allow for greater generation of plantarflexor moment with a given muscle force. However, the role of ATMA in determining ankle joint strength may require consideration of the mechanical advantage. Monte et al. (2021) investigated the role of the external moment arm and the ratio between the external to internal moment arm length (effective mechanical advantage) on hopping performance, suggesting that the ankle can adapt its leverage and mechanical function in response to different mechanical demands. In our study, we were unable to directly determine the length of the external moment arm, so we used foot length as a structural indicator, which may reflect the length of the external moment arm. However, neither foot length nor gear ratio showed any association with hopping height (Figure 4). Both ATMA and external moment arm length undergo significant changes during hopping (Monte et al., 2021), indicating that variations in length can modulate muscle-tendon interaction. Furthermore, we observed that longer foot was paired with smaller muscle mass for each muscle and in combination, as well as with thinner and shorter Achilles tendon. Similarly, we found that gear ratio exhibited similar correlations with these variables and showed a negative correlation with peak GRF (Figure 4).

Surprisingly, we did not find any relationship between muscle characteristics (such as muscle mass and ACSA and hopping performance (Figure 4). This contradicts previous reports that have demonstrated a strong connection between spatial parameters of the muscle (such as volume, mass, and ACSA) and maximal isometric and isokinetic plantarflexor strength (Bamman et al., 2000; Baxter and Piazza, 2014). Additionally, studies have shown that interindividual variation in triceps surae muscle volume can account for a significant variance in maximal plantarflexor torque (Gadeberg et al., 1999; Bamman et al., 2000; Baxter and Piazza, 2014). Based on these findings, it was reasonable to assume that larger muscle size would result in greater maximal force, which, in turn, could lead to a higher hopping height. However, contrary to our initial assumption, we could not confirm a connection between muscle size and maximal isometric force or between maximal isometric force and hopping height in our study.

Additionally, maximal hops require maximal effort, indicating that a stronger plantarflexor muscle is likely to result in higher hops. It is worth noting that the ground contact time (i.e., muscle contraction time) during hopping is shorter compared to activities such as running (Kovács et al., 2021b), jumping (Lamontagne and Kennedy, 2013; Kovács et al., 2023), or isolated isokinetic or isometric contractions in dynamometric measurements (Baxter and Piazza, 2014). This suggests that factors beyond muscle’s anatomical attributes and muscle strength, such as movement control and coordination, may contribute to the variability in hopping performance. During the push-off phase of hopping, the ATMA increases with plantarflexion, which likely facilitates tendon recoil (Farris et al., 2016). This, in turn, allows for the release of elastic energy and an increase in mechanical force output (Astley and Roberts, 2014). It has been suggested that the elastic function of the plantarflexor muscle-tendon unit is the primary source of mechanical power during hopping (Monte et al., 2021). Consequently, the mechanical characteristics of the tendon and the effective utilization of the stored strain energy within the tendon may play a more crucial role in repetitive hopping than the structural parameters of the lower leg. The major difference in our protocol compared to previous studies is that during hopping, muscles operate under a stretch shortening cycle type of contraction in oppose to only isometric or isokinetic contractile conditions (Nagano and Komura, 2003; Van Werkhoven and Piazza, 2013; Werkhoven and Piazza, 2017b; Baxter and Piazza, 2018), which can explain the different outcome. In our earlier study where we used the same task to investigate the connection between ATMA and hopping performance (Kovács et al., 2021a) but we found no direct link between hopping height and ATMA length. On the other hand, ATMA was related to peak GRF and rate of force development indicating an indirect role in muscle force production.

Our study has several methodological limitations that should be addressed. Firstly, the chosen task of repetitive bilateral hopping focused primarily on ankle thrust movement emphasizing ankle joint propulsion. However, it is important to note that knee and hip joint movements were not constrained, resulting in some degree of knee joint rotation. This may have influenced hopping performance and should be taken into consideration when interpreting the results. To mitigate this effect, hops were visually checked by an experimenter, and trials with notable knee movement were repeated. The estimation of dynamic ATMA length was performed using a polynomial model based on previous reports (Werkhoven and Piazza, 2017a). However, it is important to acknowledge that this method may not precisely represent the participants’ actual dynamic ATMA length. Nevertheless, we corrected the dynamic length using each participant’s resting ATMA length measured from MRI images, which is considered the “gold standard” method for estimating ATMA length (Fath et al., 2010). Additionally, the resting ATMA length was estimated using a two-dimensional analysis, neglecting rotations out of the plane of the image. This limitation could be overcome by implementing a more comprehensive three-dimensional determination of the moment arm. Furthermore, the inclusion of physiological cross-sectional area as a predictor of muscle force generation capacity would have allowed for a more comprehensive analysis. However, due to the lack of proper ultrasound equipment and the inability to visualize and calculate muscle fascicles, physiological cross-sectional area could not be included in the study. Incorporating this parameter would have provided a more accurate assessment of muscle force generation capacity. Lastly, male and female participants were pooled into a single group to increase the generalizability within our dataset and create a broader spectrum of the investigated variables. While there was no reason to assume that the relationships between the variables differed by gender, it is important to acknowledge this pooling of participants as a potential limitation.

In conclusion, the findings of this study suggest that longer ATMA does not lead to superior performance in a hopping task. Surprisingly, maximal isometric plantarflexion force and muscle size did not show a correlation with repetitive bilateral hopping performance. These results imply that the mechanical properties of the tendon and the efficient utilization of stored strain energy within the tendon may play a more significant role in repetitive hopping than the structural parameters of the lower leg.

## Data Availability

The raw data supporting the conclusions of this article will be made available by the authors, without undue reservation.
