# Supplementary material for: A longer Achilles tendon moment arm length is not associated with superior hopping performance
Source: Front Bioeng Biotechnol. 2023 Oct 26;11:1270169. doi: 10.3389/fbioe.2023.1270169 (PMC10639158; doi:10.3389/fbioe.2023.1270169)
Supplement: Supplementary file 2 [file DataSheet1.docx]

Supplementary Material

# Supplementary Data

# Reliability tests results:

## Achilles Tendon.

**Table 1.** Results of the ICC calculation for Achilles tendon.

| **Intraclass Correlation Coefficient** | | | | | | | |
| --- | --- | --- | --- | --- | --- | --- | --- |
|  | Intraclass Correlation^b^ | 95% Confidence Interval | | F Test with True Value 0 | | | |
|  |  | Lower Bound | Upper Bound | Value | df1 | df2 | Sig |
| Single Measures | .995^a^ | .995 | .996 | 430.212 | 709 | 709 | .000 |
| Average Measures | .998^c^ | .997 | .998 | 430.212 | 709 | 709 | .000 |
|  | | | | | | | |


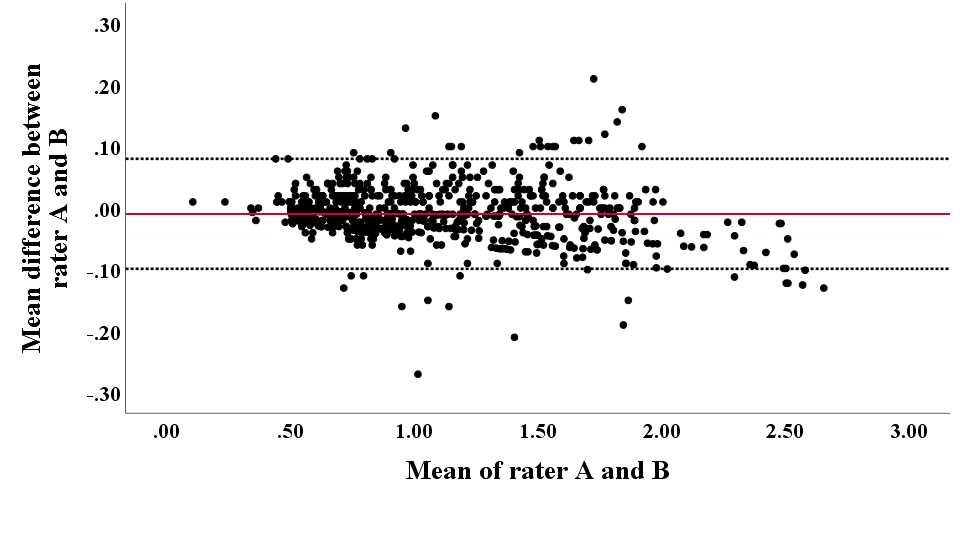


**Figure 1.** Bland-Altman plots of muscle cross-sectional area obtained from scans of the Achilles tendon. Comparisons between rater A and B are illustrated by differences between pairs of measurements as a function of the mean measurements. Solid red line depicts bias and the dotted lines depict and 95% limits of agreement.

## Soleus

**Table 2.** Results of the ICC calculation for soleus.

| **Intraclass Correlation Coefficient** | | | | | | | |
| --- | --- | --- | --- | --- | --- | --- | --- |
|  | Intraclass Correlation^b^ | 95% Confidence Interval | | F Test with True Value 0 | | | |
|  |  | Lower Bound | Upper Bound | Value | df1 | df2 | Sig |
| Single Measures | .999^a^ | .998 | .999 | 1343.890 | 1145 | 1145 | .000 |
| Average Measures | .999^c^ | .999 | .999 | 1343.890 | 1145 | 1145 | .000 |


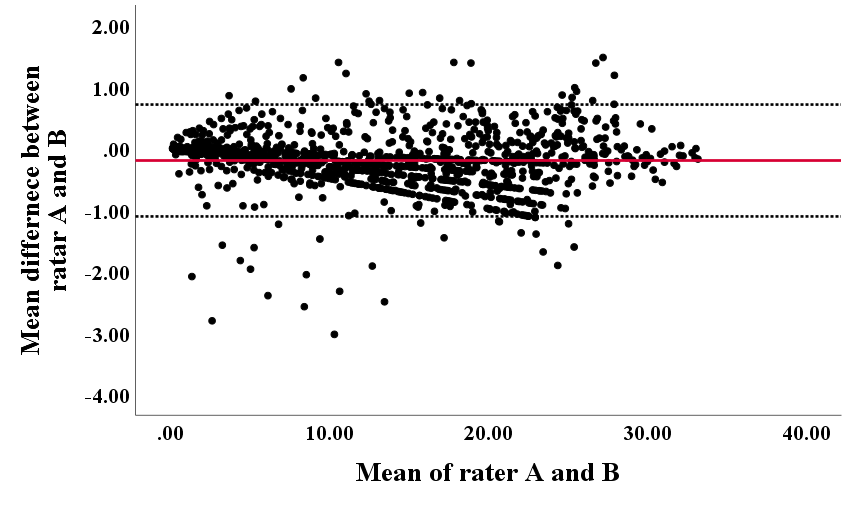


**Figure 2.** Bland-Altman plots of muscle cross-sectional area obtained from scans of the Soleus. Comparisons between rater A and B are illustrated by differences between pairs of measurements as a function of the mean measurements. Solid red line depicts bias and the dotted lines depict and 95% limits of agreement.

## Medial gastrocnemius

**Table 3.** Results of the ICC calculation for the medial gastrocnemius.

| **Intraclass Correlation Coefficient** | | | | | | | |
| --- | --- | --- | --- | --- | --- | --- | --- |
|  | Intraclass Correlation^b^ | 95% Confidence Interval | | F Test with True Value 0 | | | |
|  |  | Lower Bound | Upper Bound | Value | df1 | df2 | Sig |
| Single Measures | .998^a^ | .998 | .998 | 962.553 | 882 | 882 | .000 |
| Average Measures | .999^c^ | .999 | .999 | 962.553 | 882 | 882 | .000 |


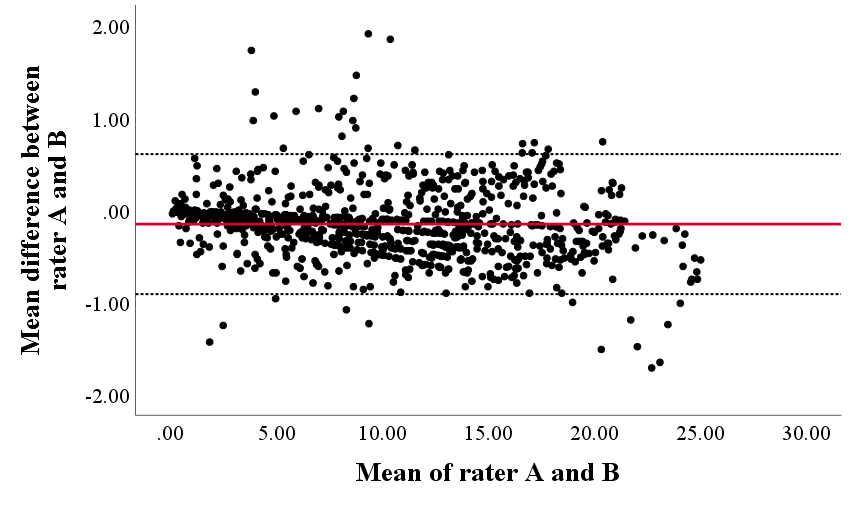


**Figure 3.** Bland-Altman plots of muscle cross-sectional area obtained from scans of the medial gastrocnemius. Comparisons between rater A and B are illustrated by differences between pairs of measurements as a function of the mean measurements. Solid red line depicts bias and the dotted lines depict and 95% limits of agreement.

## Lateral gastrocnemius

**Table 4.** Results of the ICC calculation for the lateral gastrocnemius.

| **Intraclass Correlation Coefficient** | | | | | | | |
| --- | --- | --- | --- | --- | --- | --- | --- |
|  | Intraclass Correlation^b^ | 95% Confidence Interval | | F Test with True Value 0 | | | |
|  |  | Lower Bound | Upper Bound | Value | df1 | df2 | Sig |
| Single Measures | .996^a^ | .996 | .997 | 563.254 | 766 | 766 | .000 |
| Average Measures | .998^c^ | .998 | .998 | 563.254 | 766 | 766 | .000 |


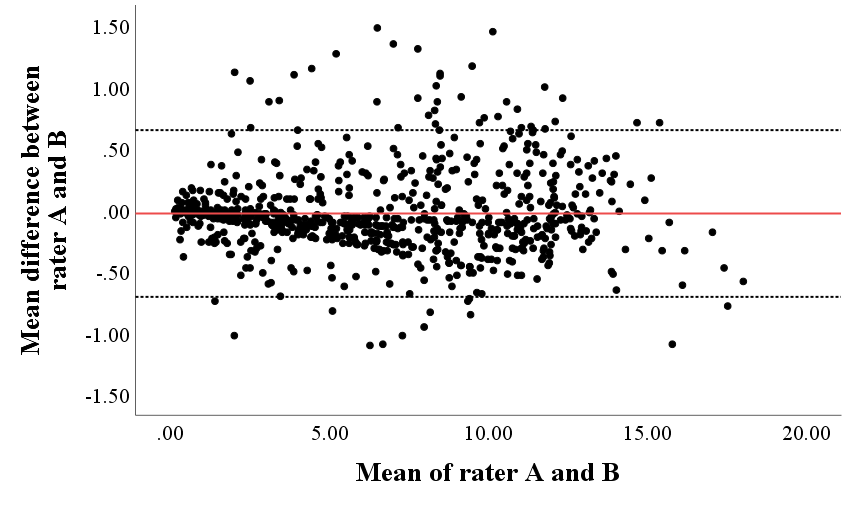


**Figure 4.** Bland-Altman plots of muscle cross-sectional area obtained from scans of the lateral gastrocnemius. Comparisons between rater A and B are illustrated by differences between pairs of measurements as a function of the mean measurements. Solid red line depicts bias and the dotted lines depict and 95% limits of agreement.
